# N^6^-Methyladenosine: A Novel RNA Imprint in Human Cancer

**DOI:** 10.3389/fonc.2019.01407

**Published:** 2019-12-19

**Authors:** Sihui Yu, Xi Li, Shiyun Liu, Rui Yang, Xiangnan Liu, Sufang Wu

**Affiliations:** ^1^Department of Obstetrics and Gynecology, Shanghai General Hospital, Shanghai Jiao Tong University School of Medicine, Shanghai, China; ^2^Department of Urology, Shanghai General Hospital, Shanghai Jiao Tong University School of Medicine, Shanghai, China

**Keywords:** epitranscriptome, RNA methylation, m^6^A, posttranscriptional control, human cancer

## Abstract

N^6^-Methyladenosine (m^6^A), a pervasive posttranscriptional modification which is reversible, has been among hotspot issues in the past several years. The balance of intracellular m^6^A levels is dynamically maintained by methyltransferase complex and demethylases. Meanwhile, m^6^A reader proteins specifically recognize modified residues and convey messages so as to set up an efficient and orderly network of m^6^A regulation. The m^6^A mark has proved to affect every step of RNA life cycle, from processing in nucleus to translation or degradation in cytoplasm. Subsequently, disorders in m^6^A methylation are directly related to aberrant RNA metabolism, which results in tumorigenesis and altered drug response. Therefore, uncovering the underlying mechanism of m^6^A in oncogenic transformation and tumor progression seeks opportunities for novel targets in cancer therapy. In this review, we conclude the extensive impact of m^6^A on RNA metabolism and highlight its relevance with human cancer, implicating the far-reaching value in clinical application.

## Introduction

N^6^-methyladenosine (m^6^A), which refers to the addition of methyl groups to the N-6 position of the adenosine residue, is a pervasive posttranscriptional RNA internal modification of eukaryotes (1). Since its first discovery in the 1970s, m^6^A had remained an uncharted territory due to technical bottlenecks (2). The stagnation ended in 2011, when the fat mass and obesity-associated protein (FTO) was revealed to exhibit demethylation activity on m^6^A-modified RNAs (3). The m^6^A mark was thus identified as a reversible process, which generated refueled passion in this field. To date, scientists have confirmed multiple m^6^A regulatory enzymes and classified them as “writers,” “erasers,” and “readers” (4).

With the availability of high-throughput sequencing technique, scientists are nowadays capable of detecting m^6^A methylation at transcriptome-wide level (5, 6). m^6^A sites are mainly enriched near stop codons, in 3′-untranslated regions (3′-UTRs) and within long internal exons. Besides messenger RNAs (mRNAs) and long non-coding RNAs, a wide range of circular RNAs (circRNAs) generated by back splice events also undergoes m^6^A modification (7). The m^6^A-circRNAs frequently arise from exons that are void of m^6^A peaks in mRNAs. This chemical mark is evolutionarily conserved and falls within a consensus motif RRACH (R = G/A, A = m^6^A, H = A/C/U) (5, 6, 8). What is more, m^6^A RNA methylation poses a broad control on RNA metabolism including alternative splicing, subcellular localization, and translational regulation (9). The impact of m^6^A regulatory enzymes on RNA processes may further interplay with tumor biology, which will be respectively discussed in this review.

## m^6^A Regulatory Enzymes Work in a Cooperative Manner

**Figure 1:**
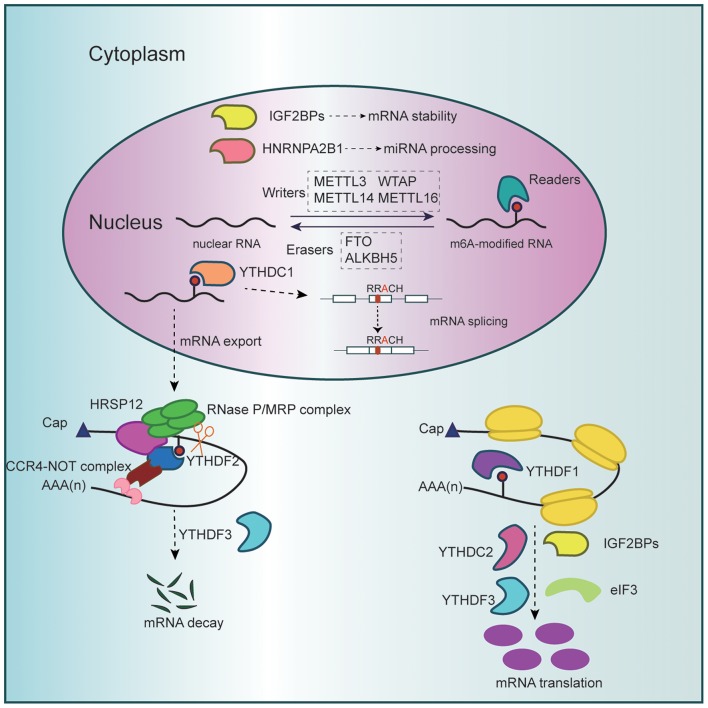
Schematic diagram of RNA m^6^A methylation. The methyltransferase complex (METTL3, METTL14, WTAP, and METTL16), which catalyzes methylation at the N6 position adenosine, and the demethylases (FTO and ALKBH5), which remove methyl groups, dynamically regulate cellular m^6^A levels in the nucleus. Besides, m^6^A modification could be specifically recognized and bound by diverse reader proteins. Nuclear m^6^A readers promote miRNA processing while affecting mRNA splicing, stability, and export. Cytoplasmic readers mediate m^6^A-marked mRNA translation and degradation.

The m^6^A regulatory enzymes work cooperatively to maintain the balance of intracellular m^6^A levels (4) (Figure 1). m^6^A “writers” composing the methyltransferase complex catalyze this modification positively. This decoration could be reversed by m^6^A “erasers” harboring demethylase activity. Meanwhile, m^6^A “readers” specifically recognize modified residues and convey messages so as to set up an efficient and orderly network of m^6^A regulation.

The m^6^A methyltransferase complex is mainly comprised of methyltransferase-like 3 (METTL3), METTL14, and Wilms' tumor 1-associating protein (WTAP), which regulate the distribution of m^6^A in coordination (10). METTL3 serves as the core component, while METTL14 is integrated with METTL3 as a stable heterodimer and catalyzes m^6^A RNA methylation through synergistic effect (10, 11). WTAP anchors METTL3–METTL14 complex to target RNAs and promotes its accumulation in nuclear speckles (8). Since WTAP harbors no methyltransferase activity, this regulatory subunit takes effect on the premise of functional m^6^A methylation complex (12). Scientists have also reported some other proteins that modulate the cellular m^6^A landscape cooperatively. METTL16, a newly defined m^6^A writer targeting U6 spliceosomal small nuclear RNA, also regulates S-adenosylmethionine homeostasis by inducing the expression of S-adenosylmethionine synthetase upon methionine starvation (13–16).

The m^6^A erasers discovered so far involve two candidates, FTO and AlkB homolog 5 (ALKBH5). FTO was initially proved to regulate energy homeostasis and is positively related to risk of obesity (17, 18). ALKBH5 is a homolog of FTO, and they both belong to the Fe(II)- and oxoglutarate-dependent AlkB oxygenase family (17–19). In the m^6^A circuit, FTO and ALKBH5 identify m^6^A-modified nuclear RNAs as substrate and catalyze removal of m^6^A mark (3, 19).

The reader proteins specifically recognize m^6^A decoration to sort mRNAs for quicker metabolism to further perform biological functions (4). Among these readers, YT521-B homology (YTH) domain proteins are the best documented, including YTH domain family proteins (YTHDF1–3) and YTH domain containing proteins (YTHDC1–2) (20–25). YTHDF1–3 and YTHDC2 are cytoplasmic readers, while YTHDC1 mainly operates in the nucleus. Insulin-like growth factor 2 mRNA-binding proteins (IGF2BP1–3) is a distinct family of readers with K homology (KH) domains to recognize m^6^A (26). More potential m^6^A readers are under exploration, such as heterogeneous nuclear ribonucleoprotein A2/B1 (HNRNPA2B1) and eukaryotic initiation factor 3 (eIF3) (27, 28).

## m^6^A Regulates RNA Metabolism in Physiological Conditions

The RNA life cycle comprises RNA processing, export, and translation or degradation. Formidable evidence has shown that m^6^A and its regulatory enzymes take part in every step of RNA metabolism. Generally speaking, the writers and erasers dictate m^6^A levels in specific targets, which are decoded by the readers to accelerate RNA process and translate into distinct functions.

### RNA Processing in Nucleus

WTAP favors the positioning of METTL3–METTL14 complex in nuclear speckles that are sites associated with RNA processing and transcription (10). As a result, WTAP modulates alternative splicing and gene expression. Similarly, ALKBH5 colocalizes with nuclear speckles, and m^6^A erasure mediated by ALKBH5 is critical for correct splicing, preventing longer 3′-UTR mRNAs from quick degradation during spermiogenesis (29).

YTHDC1 binds m^6^A-modified pre-mRNAs and affects RNA binding affinity of splicing factors (30). Under normal circumstances, YTHDC1 promotes mRNA binding of SRSF3 while antagonizing that of SRSF10, predominantly triggering exon inclusion of targeted mRNAs (31) (Figure 1). In addition, YTHDC1 interacts with pre-mRNA 3′ end processing factors such as CPSF6 and determines the length of 3′-UTR where lie many microRNAs (miRNAs) target sites (25). Given that miRNAs pair to mRNAs of protein-coding genes and repress them at posttranscriptional level, YTHDC1 regulates mRNA stability and translation efficiency indirectly (25, 32).

Meanwhile, m^6^A mark induced by METTL3 on primary miRNAs could be recognized by adaptor readers such as HNRNPA2B1 (27, 33). The microprocessor protein DGCR8 is then recruited to specific precursor miRNAs and encourages their processing into mature miRNAs.

### mRNA Export From Nucleus to Cytoplasm

In addition to alternative splicing and exon inclusion, YTHDC1 favors export of methylated mRNAs from nucleus to cytoplasm by the aid of SRSF3, indirectly triggering translation via increased cytoplasmic abundance of targets (34) (Figure 1).

Consistently, *Alkbh5* deficiency in male mice lifts m^6^A levels and facilitates mRNA export to cytoplasm (35, 36). Cytoplasmic levels of mRNAs critical for proper spermatogenic maturation are altered, leading to aberrant spermatogenesis and apoptosis. Thus, it appears that m^6^A exerts complex roles on subsequent effect of mRNA export. In addition, the latest findings show that Fragile X mental retardation protein, a newly identified m^6^A reader protein, is also capable of facilitating the nuclear export of m^6^A-marked transcripts through directly binding to a collection of m^6^A sites on target mRNAs (37).

### mRNA Translation or Degradation in Cytoplasm

After nuclear export, m^6^A-modified RNAs will be sorted into different groups depending on diverse readers and then undergo a fast-tracking metabolism for translation or dedicated degradation. This process helps to generate adequate protein for urgent demand or rapidly degrade mRNAs in necessity (2).

YTHDF1 binds m^6^A-modified RNAs and promotes ribosome loading via recruiting initiation factors (eIFs) that are pivotal in the rate-limiting step of translation (20) (Figure 1). Accordingly, YTHDF1 facilitates translation efficiency and protein synthesis. Other readers including YTHDC2, IGF2BPs, and eIF3 also bind m^6^A at its consensus motif to enhance translation efficiency (23, 24, 26, 28).

On the contrary, YTHDF2 mediates m^6^A-containing RNA decay, thus regulating gene expression and cell fate (21). YTHDF2 knockdown results in accumulation of untranslated target mRNAs, thereby reducing translation efficiency. Specifically speaking, carboxy-terminal domain of YTHDF2 selectively binds to m^6^A-modified mRNA, while amino-terminal domain localizes the YTHDF2–mRNA complex to RNA decay sites. Recently, the molecular mechanism underpinning YTHDF2-directed RNA decay has been expounded. m^6^A-containing linear and circular RNAs undergo endoribonucleolytic cleavage through YTHDF2-HRSP12-RNase P/MRP axis, coupled to CCR4-NOT complex-mediated deadenylating pathway (38) (Figure 1). In addition, a recent study reported that multivalent m^6^A-modified RNAs could promote the phase separation of YTHDFs and that phase separation of m^6^A and YTHDF2 might participate in cellular response to stresses, despite the uncertainty of its specific role (39).

After nuclear export of methylated RNAs, YTHDF3 tunes their delivery before YTHDF1 and YTHDF2, especially partitioning their shared targets (22). YTHDF3 accelerates translation or decay of m^6^A-containing mRNAs in synergy with YTHDF1 and YTHDF2. By the way, a broad range of circular RNAs generated by pre-mRNA back splicing in human transcriptome has coding potential and bears m^6^A modification (40). YTHDF3 drives protein translation from these circRNAs in a cap-independent fashion.

Except for the YTHDF family members, SND1, a putative m^6^A reader of the “royal family” also binds to m^6^A modification of *ORF50* RNA and stabilizes the transcript, which favors the replication of Kaposi's sarcoma-associated herpesvirus (41).

## m^6^A Poses Control on Tumorigenesis and Cancer Progression

Researchers have involved m^6^A decoration in the development of human diseases. Mechanistically, m^6^A could alter the expression of mRNAs encoding various regulators such as transcription factors and function as either barrier or facilitator of malignant transition in tumor cells. In this section, we respectively state the variable roles of m^6^A in tumorigenesis and cancer progression based on different m^6^A regulatory enzymes (Table 1).

**Table 1:** The summary of roles of m^6^A subunits in tumor formation and progression.

**Candidate**	**Tumor tissues or cell lines**	**Function**	**Mechanism**	**References**
METTL3 and METTL14	Glioblastoma stem cells (GSCs)	Tumor suppressor	Reduces oncogene (*ADAM19, EPHA3, KLF4*) and upregulates tumor suppressor (*CDKN2A, BRCA2, TP53I11*) expression, inhibits GSC growth, self-renewal *in vitro*, and glioblastoma progression *in vivo*	(42)
METTL3	Glioma stem-like cells (GSCs)	Oncogene	Enhances *SOX2* mRNA stability, contributes to efficient DNA repair and GSC maintenance, promotes tumor propagation *in vivo*	(43)
METTL3	Pancreatic cancer cells	Oncogene	Induces resistance to anticancer reagents such as GEM, 5-fluorouracil, cisplatin, and irradiation	(44)
METTL3	Breast cancer tissues	Oncogene	Lifts expression of *HBXIP*, accelerates cell proliferation, and inhibits apoptosis	(45)
METTL3	Bladder cancer tissues and cell lines	Oncogene	Promotes *CDCP1* translation and inhibits *PTEN* expression through positively modulating pri-miR221/222 process, enhances cell proliferation, invasion, and survival *in vitro* and *in vivo*	(46, 47)
METTL3	Lung adenocarcinoma tissues	Oncogene	Recruits eIF3 to translation initiation complex, promotes translation of oncogenes including *BRD4, EGFR* and the Hippo pathway effector *TAZ*, enhances cell growth, survival, and invasion	(48, 49)
METTL3	Acute myeloid leukemia cells	Oncogene	Enhances translation of *c-MYC, BCL2*, and *PTEN* mRNAs, blocks cell differentiation and apoptosis, promotes leukemia progression	(50, 51)
METTL3	Hepatocellular carcinoma tissues	Oncogene	Destabilizes *SOCS2* mRNA through YTHDF2-mediated degradation, enhances HCC growth and metastasis, indicates poor prognosis of HCC	(52)
METTL3 and METTL14	Endometrial tumor tissues	Tumor suppressor	Upregulates PHLPP2 expression and downregulates mTORC2 expression, attenuates AKT activity, inhibits cell proliferation, migration, and *in vivo* tumor growth	(53)
METTL14	Hepatocellular carcinoma tissues	Tumor suppressor	Enhances recognition of pri-miR126 by DGCR8 and processing to mature miRNA, suppresses tumor metastasis *in vitro* and *in vivo*	(54)
METTL14	Acute myeloid leukemia cells	Oncogene	Enhances stability and translation of *MYB* and *MYC* mRNA, blocks myeloid differentiation, contributes to maintenance and self-renewal of LSCs/LICs	(55)
WTAP	Renal cell carcinoma tissues and cell lines	Oncogene	Stabilizes the transcript and promotes *CDK2* expression, enhances cell proliferation *in vitro* and tumorigenesis *in vivo*, indicates poor prognosis	(56)
WTAP	Pancreatic cancer	Oncogene	Stabilizes *Fak* mRNA, activates Fak-PI3K-AKT and Fak-Src-GRB2-Erk1/2 pathways, promotes migration/invasion both *in vitro* and *in vivo*	(57)
FTO	Glioblastoma stem cells (GSCs)	Oncogene	Induces expression of oncogenes (*ADAM19, EPHA3, KLF4*), promotes GSC growth, self-renewal *in vitro* and brain tumor development *in vivo*	(42)
FTO	Breast cancer tissues and cell lines	Oncogene	Induces degradation of *BNIP3* mRNA, bursts tumor growth and metastasis *in vitro* and *in vivo*, suggests poor clinical outcome	(58)
FTO	Acute myeloid leukemia cells	Oncogene	Represses expression of *ASB2* and *RARA*, enhances cell proliferation *in vitro*, promotes leukemogenesis *in vivo*, blocks ATRA-induced cell differentiation	(59)
FTO	Acute myeloid leukemia cells	Oncogene	Increases *MYC/CEBPA* transcript levels and associated pathways, promotes leukemia cell proliferation/viability *in vitro*, enhances AML progression *in vivo* and shrinks mice survival	(60)
FTO/ALKBH5	*BRCA*-mutated epithelial ovarian cancer cells	Tumor suppressor	Destabilizes *FZD10* mRNA, inhibits Wnt/β-catenin, enhances cell sensitivity to PARP inhibitors	(61)
ALKBH5	Hypoxic breast cancer cells	Oncogene	Stabilizes *NANOG* mRNAs, induces breast cancer stem cell (BCSC) enrichment, promotes tumor initiation	(62)
ALKBH5	Glioblastoma stem-like cells (GSCs)	Oncogene	Enhances *FOXM1* expression, promotes GSCs proliferation *in vitro* and tumorigenesis *in vivo*	(63)
YTHDF1	Colorectal cancer tissues	Oncogene	Promotes cell proliferation, enhances resistance to fluorouracil and oxaliplatin	(64)
YTHDF1	Ocular melanoma	Tumor suppressor	Promotes the translation of *HINT2* mRNA, inhibits tumor progression *in vitro* and *in vivo*	(65)
YTHDF1	Nonsmall cell lung cancer cells	Oncogene/tumor suppressor	Promotes translation of CDK–cyclin complex and enhances tumor growth under normoxia condition; sensitizes cancer cells to cisplatin through reduced Nrf2-AKR1C1, the clearance system of reactive oxygen species (ROS)	(66)
YTHDF2	Hepatocellular carcinoma cells	Tumor suppressor	Promote degradation of *EGFR* mRNA, inhibits extracellular-signal-regulated kinase/mitogen-activated protein kinase signaling, suppresses cell proliferation and tumor growth *in vitro* and *in vivo*; represses inflammation and vascular abnormalization *via IL11* and *SERPINE2* mRNA decay, promotes metastasis	(67, 68)
YTHDF2	Acute myeloid leukemia cells	Oncogene	Downregulates TNFR2, facilitates LSC development and AML propagation	(69)
YTHDC2	Colon cancer tissues	Oncogene	Facilitates translation of *HIF-1α* and *Twist1* mRNA in hypoxia, promotes cancer metastasis	(70)
IGF2BP1-3	Cervical and liver cancer cells	Oncogene	Enhances mRNA stability and translation, upregulates oncogenic genes such as *MYC*, facilitates tumor growth and invasiveness	(26)
IGF2BP1	Ovarian, liver, and lung cancer cells	Oncogene	Impairs miRNA-directed decay of *SRF* mRNA, enhances serum response factor (SRF)-driven transcription, sustains expression of *PDLIM7*, and *FOXK1*, promotes tumor growth and invasion	(71)
IGF2BP2	Colorectal tumor tissues	Oncogene	Stabilizes *HMGA2* mRNA by forming a circNSUN2/IGF2BP2/*HMGA2* ternary complex, promotes colorectal liver metastasis both *in vitro* and *in vivo*	(72)

### m^6^A Writers

#### METTL3

In glioblastoma stem cells (GSCs), Cui et al. knocked down METTL3 to hinder m^6^A enrichment, and they also observed enhanced growth, self-renewal of GSCs, and tumor progression (42). In this process, oncogenes such as *ADAM19, EPHA3*, and *KLF4* were upregulated, while expression of tumor suppressors involving *CDKN2A, BRCA2*, and *TP53I11* were impeded. On the contrary, another study argued that METTL3 plays an oncogenic role in glioblastoma via methylating 3′-UTR of *SOX2* mRNA, which encodes transcription factors enabling the regain of stem-like properties and efficient DNA repair (43). The m^6^A modification enhances the stability of *SOX2* mRNA. Accordingly, silencing of METTL3 interrupts SOX2-dependent DNA repair, impairs GSC maintenance, and delays tumor propagation *in vivo*. Different target mRNAs of m^6^A mark, genetic, and non-genetic heterogeneity of cancer stem cells (CSCs) shall account for the controversy. Studies in normal stem cells have also been performed to complement the results, which are quite different. In adult neural stem cells, depletion of METTL3 reduces m^6^A levels on transcripts of histone methyltransferase *EZH2* and inhibits its protein expression (73). Scientists reported that m^6^A depletion not only suppressed cell growth but also blocked neuronal development and morphological maturation. This conclusion also implicates certain crosslink between m^6^A mark and histone modification.

Besides, additional studies have verified the role of METTL3 in oncogenic transformation of various tumors. For instance, METTL3 depletion sensitizes pancreatic cancer cells to anticancer agents such as gemcitabine, 5-fluorouracil, cisplatin, and irradiation (44).

The METTL3-induced m^6^A mark also drives malignant progression in breast tumor in aid of hepatitis B X-interacting protein (*HBXIP*), an oncogene in breast cancer cells (45). METTL3 lifts the mRNA and protein levels of *HBXIP*, which in turn promotes the expression of METTL3 and forms a positive feedback loop. The m^6^A regulation in mRNA stability could have a bearing on this procedure.

Scientists have also reported in bladder cancer that METTL3 accelerates the processing of pri-miR221/222 via recognition by DGCR8 (46). Subsequently, mature miR221/222 restrains the expression of the antioncogene *PTEN* and ultimately boosts tumor growth both *in vitro* and *in vivo*. Based on the preferential m^6^A recognition by YTHDF1, METTL3 also facilitates translation of oncogene *CDCP1*, which plays a pivotal role in bladder cancer progression (47). Simultaneously, this biological process exerts synergistic effect with chemical carcinogens in malignant transformation of uroepithelial cells.

In human lung cancer, gain-of-function study of METTL3 motivates cell growth and invasion, giving rise to tumors of larger size in mouse xenografts (48, 49). METTL3 was found to bind m^6^A sites near the stop codon of specific mRNAs and recruit eIF3 to translation initiation complex in the 5′ end, which mediates mRNA circularization and ribosome recycling. In this way, METTL3 directly promotes efficient translation of onco-proteins involving BRD4, EGFR, and TAZ. Notably, the methyltransferase activity and m^6^A-binding readers are proved to be uncoupled. This finding proposes a novel model of METTL3 in translational control, and the molecular determinants, such as the specificity of target mRNAs and localization of m^6^A peaks, are worth in-depth investigation.

In acute myeloid leukemia cells (AMLs), the abundance of METTL3 is elevated compared to that in normal hematopoietic stem/progenitor cells (HSPCs) (50). Cell proliferation is inhibited along with depletion of this enzyme, and leukemogenesis is also delayed *in vivo*. Besides, METTL3 level is negatively relevant to the status of differentiation and apoptosis in AML cells. Inactivation of AKT induced by METTL3 overexpression contributes partially to the block of differentiation in an m^6^A-independent manner. Further research suggests that METTL3 promotes the translation of functional proteins regulating cell cycle progression and apoptosis, such as c-MYC and BCL2. A later study instructed that METTL3 is recruited by the CAATT-box binding protein CEBPZ to promoters of active genes and mediates m^6^A methylation within coding regions of target transcripts (51). Translation of genes necessary for AML is thus enhanced via relieved ribosome stalling. As a result, an alternative mechanism of METTL3 in translational regulation has been put forward.

In hepatocellular carcinoma (HCC), METTL3 is significantly upregulated and indicates poor prognosis (52). Mechanistically, METTL3 promotes HCC growth and invasiveness by repressing the expression of suppressor of cytokine signaling 2 (*SOCS2*), a tumor suppressor in HCC, through m^6^A-YTHDF2-dependent mRNA degradation.

Nevertheless, expression of METTL3 is reduced in endometrial carcinoma, which stimulates AKT signaling and promotes tumor growth and invasiveness both *in vitro* and *in vivo* (53). Mechanistically, lower expression of METTL3 reduces m^6^A methylation, restrains YTHDF1-promoted translation of PHLPP2, a negative AKT regulator, while dampens YTHDF2-promoted decay of transcripts encoding mTORC2, which is a positive AKT regulator.

In a nutshell, METTL3 modulates the expression of oncogenes and tumor suppressor genes primarily at posttranscriptional levels, including mRNA stability and translational process. Consequently, different downstream targets of METTL3 and the dominant cancer-related pathways involved in the process bring about the discrepancy in cell fate of different tumors.

#### METTL14

As is the case of METTL3 in glioblastoma, METTL14 depletion facilitates the malignant phenotype, characterized by upregulated oncogenes such as *ADAM19* and reduced expression of tumor suppressors such as *CDKN2A* (42). Meanwhile, loss-of-function mutation of METTL14 in endometrial tumor also diminishes m^6^A methylation, inhibits YTHDF1-mediated translation of PHLPP2, and impedes YTHDF2-related mRNA decay of mTORC2, both of which regulate AKT pathway, as aforementioned in METTL3 (53). Subsequently, cell proliferation and tumorigenicity of endometrial tumors are increased, along with AKT stimulation.

Furthermore, m^6^A modification is suppressed in HCC tissues, and METTL14 downregulation suggests poor prognosis for recurrence-free survival (54). In HCC, METTL14 restrains metastasis by enhancing pri-miR126 process into mature miRNA in a DGCR8-dependent manner. This result is opposite to the conclusion drawn by Chen et al. in primary HCC tissues that m^6^A is significantly increased and overexpression of METTL3 promotes liver carcinogenesis through m^6^A-YTHDF2-dependent degradation of *SOCS2* mRNAs (52). The controversy may be attributed to complex factors, including different reader proteins to sort mRNA transcripts, as well as distinct tumor samples and methodology of m^6^A detection.

However, the opposite conclusion has been drawn in hematopoietic diseases. In normal HSPCs and AML cells carrying t(11q23), t(15;17), or t(8;21), METTL14 is overexpressed and exerts oncogenic role through m^6^A signal by positively manipulating the stability and translation of *MYB* and *MYC* mRNA (55). This result is partially overlapped with the impact of METTL3 in AML, and might be explained by alternative reading process mediated by IGF2BPs, for an example. METTL14 undertakes an essential role in self-renewal of leukemia stem/initiation cells (LSCs/LICs) and AML progression (55). Silencing of METTL14 facilitates differentiation of both normal HSPCs and AML cells while repressing AML cell survival.

#### WTAP

In cancerous tissues of glioblastoma and cholangiocarcinoma, WTAP is overexpressed and promotes cell migration and invasion (74, 75). However, the regulation WTAP exerts on cell proliferation is cell-type specific. In AML, WTAP supports tumor growth but arrests differentiation of leukemia cells (76).

In renal cell carcinoma, WTAP indicates poor survival of patients, and knockdown of WTAP impedes cell proliferation *in vitro* and tumorigenesis *in vivo* (56). Mechanistically, WTAP binds to 3′-UTR of cyclin-dependent protein kinase 2 (*CDK2*) mRNAs and stabilizes the transcripts, lifting CDK2 protein level. As a key regulator of cell cycle, upregulation of CDK2 enables cell to cross the G1/S limit and initiates DNA replication. Similarly, in pancreatic cancer, WTAP promotes cell migration, invasion, and chemoresistance to gemcitabine via stabilizing focal adhesion kinase (*Fak*) mRNA and subsequently activating Fak-PI3K-AKT and Fak-Src-GRB2-Erk1/2 pathways (57).

To the best of our knowledge, WTAP promotes tumorigenic change in a variety of tumors. However, the underlying mechanism remains elusive. Future researches are required to unveil whether the regulatory role of WTAP in m^6^A decoration is linked to these biological processes.

### m^6^A Erasers

#### FTO

A number of studies have attested to the tumorigenic role of FTO in various sorts of cancers. In endometrial carcinoma, β-estradiol induces expression of FTO and mediates cell growth and invasion (77). In addition, FTO inhibitor has been reported to abolish the expression of oncogenes such as *ADAM19* and to suppress GSC growth, self-renewal *in vitro*, and tumor development *in vivo* (42).

Silencing of FTO also attenuates cell growth and metastasis in breast cancer (58). Mechanistically, FTO disturbs the expression of *BNIP3*, a proapoptotic gene, both in mRNA and protein levels, via demethylating m^6^A residues in 3′-UTR. On the other hand, YTHDF2 binding has proved to be uncoupled.

FTO is also significantly overexpressed in AMLs with t(11q23)/MLL rearrangements, t(15;17)/PML-RARA, FLT3-ITD, or NPM1 mutations (59). Reducing m^6^A levels in *ASB2* and *RARA* mRNAs, FTO destabilizes the transcripts and, as a result, enhances leukemogenesis while blocks cell differentiation induced by all-trans-retinoic acid (ATRA) in these AML subtypes. Besides, researches also precluded YTHDF1/2 as readers regulating the stability of *ASB2* and *RARA* mRNAs.

Interestingly, R-2-hydroxyglutarate (R-2HG), a metabolic product in isocitrate dehydrogenase mutant cancers such as AML, is similar to α-KG structurally and competitively represses Fe (II)/α-KG-dependent dioxygenases (60). Thus, FTO could be suppressed by R-2HG in sensitive leukemia cells to elevate global m^6^A RNA modification, which destabilizes the *MYC/CEBPA* transcripts and reduces their expression. As a crucial transcription factor in leukemogenesis, CEBPA being inhibited further inactivates FTO as a feedback loop and reinforces the growth-suppressive effect. Compared with METTL14 which promotes *MYC* mRNA stability via modulating m^6^A abundance on 3′-terminal exons, FTO enhances MYC expression by demethylating m^6^A sites on 5′-terminal and internal exons, which inhibits the YTHDF2-mediated RNA decay (55, 60).

However, in epithelial ovarian cancers (EOC) with *BRCA* mutation, downregulation of FTO confers resistance to PARP inhibitors such as Olaparib, with m^6^A enrichment in 3′-UTR regions of *FZD10* and increased mRNA stability (61). FZD10 positively upregulates Wnt/β-catenin pathway and further promotes activity of homologous recombination. Meanwhile, stabilization of *FZD10* mRNA is mainly caused by the predominant effect of IGF2BP2, also overexpressed in resistant cells.

Obviously, when FTO mediates deprivation of m^6^A signaling that is previously recognized by readers promoting mRNA stability, corresponding mRNA levels would be impaired. On the contrary, protein-coding mRNAs would be upregulated if FTO prevents YTHDF2-mediated mRNA decay via m^6^A erasure. In this way, different binding proteins and downstream targets regulate the trend of tumor growth in coordination.

#### ALKBH5

Similar to FTO in *BRCA*-mutated EOC, expression of ALKBH5 is also inhibited, which activates Wnt/β-catenin pathway via stabilizing *FZD10* mRNA and renders cell resistance to Olaparib (61).

However, all sites subject to m^6^A modification are not equally critical, since they are chosen to be involved in different biological pathways. ALKBH5 may play distinct roles from FTO due to their preference in molecular substrates. In hypoxic breast cancer cells, ALKBH5 demethylates *NANOG* mRNA and elevates the protein level via reduced mRNA decay, on the premise of hypoxia-inducible factors (HIFs) (62). As a pluripotency factor, upregulation of NANOG leads to enrichment of breast CSCs (BCSCs). Otherwise, ALKBH5 knockdown inhibits NANOG expression, reduces BCSC population, and impairs tumor formation *in vivo*.

Similarly, in glioblastoma, GSCs proliferation and tumor formation is disrupted upon ALKBH5 inhibition (63). Owing to enzymatic activity of ALKBH5, the nascent transcripts encoding FOXM1, a transcription factor, are stabilized and thus increases expression of relevant protein. Besides, interplay between ALKBH5 and FOXM1 can be enhanced by a non-coding RNA antisense to FOXM1 (*FOXM1-AS*).

### m^6^A Readers

#### YTHDF1

In colorectal cancer tissues, c-Myc drives the expression of YTHDF1 transcriptionally, and high level of YTHDF1 suggests poor prognosis in patients (64). Knockdown of YTHDF1 hinders cell proliferation and renders sensitization to fluorouracil and oxaliplatin. However, the detailed mechanism remains unknown.

Notably, YTHDF1 recognizes m^6^A-marked transcripts of lysosomal proteases and promotes translation of lysosomal cathepsins in dendritic cells, which favors antigen degradation (78). Cross-presentation of engulfed neoantigens and cross-priming of CD8^+^ T cells are then suppressed, contributing to the immune evasion and incomplete tumor elimination.

On the other hand, in ocular melanoma, YTHDF1 promotes the translation of m^6^A-containing *HINT2* mRNA, a tumor suppressor (65). Scientists reported decreased m^6^A levels in these tumor samples, which was significantly correlated with tumor progression both *in vitro* and *in vivo*. Therefore, specific m^6^A-modified targets of YTHDF1 might vary according to the cellular context, resulting in different functions of YTHDF1 in various tumors.

Interestingly, a recently released study demonstrated the critical and contradictory role of YTHDF1 in hypoxia adaptation and pathogenesis of non-small cell lung cancer (66). Under normoxia conditions, YTHDF1 depletion restrains non-small cell lung cancer tumor growth *in vitro* and *in vivo*, which resulted from reduced translational efficiency of m^6^A-marked transcripts such as CDK2, CDK4, and cyclin D1. On the other side, YTHDF1 deficiency renders resistance of cancer cells to cisplatin and indicates poor clinical outcome. Further study revealed that, under chemotherapy stress condition, YTHDF1 depletion leads to decreased translation of m^6^A-modified Keap1, which upregulates Nrf2 and AKR1C1, the clearance system of reactive oxygen species. The adverse results highlight the importance of achieving a homeostasis of YTHDF1 expression and its targets between normal and stressful conditions.

#### YTHDF2

In HCC cells, YTHDF2 can be specifically restricted by hypoxia and act as a tumor suppressor with inhibitory effect on tumor growth (67). Mechanistically, YTHDF2 directly binds m^6^A sites in 3′-UTR and mediates the degradation of *EGFR* mRNA, which is a main upstream regulator of extracellular-signal-regulated kinase/mitogen-activated protein kinase pathway. Hou et al. have also revealed in HCC that YTHDF2 reduction provokes inflammation and vascular reconstruction, which facilitates the progression of tumor metastasis (68). In detail, the YTHDF2-mediated decay of m^6^A-containing mRNAs are disrupted, such as interleukin 11 (*IL11*) and serpin family E member 2 (*SERPINE2*), which are account for the inflammation-associated malignancy and vascular abnormalization. What is more, administration of PT2385, a small molecule inhibitor targeting HIF-2α and restoring the expression of YTHDF2, also exhibits favorable effects in treating HCC cells both *in vitro* and *in vivo*.

Moreover, the roles of YTHDF2 in different context mainly depend on the degradation of respective target mRNAs. Paris et al. reported that YTHDF2 shortens half-life of m^6^A-modified mRNAs of TNF receptor 2 (*TNFR2*), which normally prevents accumulation of leukemic cells and thus facilitates AML propagation (69). Targeting YTHDF2 not only eradicates LSCs but also expands hematopoietic stem cells (HSCs) to enhance myeloid reconstitution. In consequence, YTHDF2 inhibitor is considered as a candidate strategy for AML treatment. A noteworthy phenomenon in biological condition is that YTHDF2 mediates clearance of m^6^A-modified mRNAs of Wnt-related genes to suppress Wnt signaling at stable state and maintain HSC quiescence (79). Upon hematological stresses, downregulation of YTHDF2 aberrantly upregulates target genes of Wnt signaling as well as survival-associated genes, which elevates not only proliferation but also regeneration capacity of HSCs synergistically, as a protective measure. Thus, we could gain a better understanding of the dual character of YTHDF2 in stem cells under physiological and pathological conditions.

#### YTHDC2

In colon cancer tissues, expression of YTHDC2 is positively correlated with the tumor stage (70). Further research shows that YTHDC2 unwinds highly structured 5′-UTR of mRNAs encoding transcription factors, HIF-1α and Twist1, and facilitates their translation. Notably, HIF-1α promotes epithelial-to-mesenchymal transition via the key regulator Twist1, initiating tumor metastasis.

#### IGF2BPs

IGF2BPs, a group of direct m^6^A-binding proteins, enhance mRNA stability and translation both under normal and stress conditions, which gives rise to accumulation of oncogenic products such as MYC (26). In the absence of IGF2BPs, cell proliferation and invasion are significantly repressed in cervical and liver cancer cells.

IGF2BP1 impairs miRNA-directed degradation of mRNAs and sustains expression of serum response factor in ovarian, liver, and lung cancers, potentially in an m^6^A dependent manner (71). This process enhances serum response factor-driven transcription and upregulates oncogenic drivers such as PDLIM7 and FOXK1.

Furthermore, IGF2BP2 has recently been proven to mediate colorectal liver metastasis, testified both *in vitro* and *in vivo* with metastasis PDX models (72). Mechanistically, the m^6^A modification of circNSUN2 is recognized by YTHDC1, which accelerates the cytoplasmic export and further stabilizes *HMGA2* mRNA by forming a circNSUN2/IGF2BP2/*HMGA2* ternary complex in the cytoplasm. This outcome suggests a brand-new role of IGF2BP2 in mRNA stabilization via an m^6^A-independent way and provides evidence that m^6^A-modified circRNAs could serve as prognostic markers.

## Bioinformatics: An Emerging Series of Tools for m^6^A Exploration

Meanwhile, with the field of bioinformatics booming in the past several years, researchers have established a number of databases delineating m^6^A machinery, which provides valuable and comprehensive clues for future study (80–84). For instance, RMBase v2.0 deciphers the landscape of RNA modifications based upon epitranscriptome-sequencing data, while MODOMICS provides information regarding RNA modification pathways (80, 81).

Certainly, there exist multiple databases dedicated to the improvement of the m^6^A-associated knowledge. To take MeT-DB v2.0 as an example, a powerful platform for methyl-transcriptomic research, identifies m^6^A peaks as well as single-base sites (82). More importantly, context-specific functions of m^6^A are elucidated via peak distribution plot and gene expression profiles under different conditions to identify m^6^A-driven genes and networks. Another database, m6AVar, allows annotation and visualization of functional variants in the vicinity of m^6^A sites and helps interpret their impact on m^6^A mark by converting RNA sequences of target sites or key flanking nucleotides (83). This database also incorporates data from genome-wide association studies and ClinVar to identify disease-causing variants and explore their pathogenic molecular mechanisms. Both of the two databases intersect m^6^A-modified sites with functional data such as binding sites of RNA-binding proteins and splicing factors as well as miRNA target sites to obtain regulatory pairs and speculate their roles in posttranscriptional regulation (82, 83).

In addition, a study has recently reported the molecular feature and clinical relevance of m^6^A regulators reconstituted across 33 cancer types (84). The authors found widespread genetic alterations (mutations and copy number variations) to m^6^A enzymes and established the cross-talk between their expression patterns with activity of cancer hallmark-related pathways, putatively helpful in prognostic stratification. Thus, we could see that bioinformatic tools not only complements the experimental results but also expedites the discovery of unrecognized regulatory roles of m^6^A mark.

## Clinical Relevance of m^6^A-Targeted Strategy

So far, small-molecule inhibitors targeting m^6^A regulatory enzymes are not available in clinical use. However, due to the tumorigenic role of FTO in various cancers, scientist have developed several FTO inhibitors as promising tools in antileukemia and antiglioblastoma therapies.

As mentioned above, R-2HG exhibits broad antiproliferative effects in high-FTO leukemia via targeting FTO/MYC/CEBPA signaling (60). Meanwhile, R-2HG also has synergistic effect with first-line chemotherapy drugs such as decitabine and daunorubicin, which was validated in mouse models. Later on, Huang et al. utilized structure-guided design and developed two small-molecule FTO inhibitors, FB23 and its derivative FB23-2 (85). In comparison, the latter shows significantly improved antiproliferative activity in AML cells and induces cell differentiation. The authors also observed delayed AML progression and prolonged survival *in vivo*, which enlightens the strategy of targeting FTO demethylase in AML treatment.

Meclofenamic acid (MA) is originally approved by the Food and Drug Administration as a non-steroidal anti-inflammatory drug (86). MA2, which refers to the ethyl ester form of MA, has been identified as a selective FTO inhibitor, increasing m^6^A levels in mRNAs. Application of MA2 represses GSC-initiated tumor progression and extends lifespan of xenografted mice (42).

In addition, scientist have newly identified entacapone, an inhibitor of catechol-O-methyltransferase applied for treatment of Parkinson's disease, as a chemical FTO inhibitor (87). Entacapone elicits effects on metabolic homeostasis through selectively targeting FTO activity, whereas its function in tumorigenesis remains to be elucidated.

Novel anticancer agents targeting other m^6^A enzymes could possibly have therapeutic value as well. For example, METTL14 inhibitors are likely to be effective strategies to treat specific AML subtypes with high METTL14 expression, especially in combination with standard agents that induce myeloid differentiation (55). Combinatorial treatment of METTL3 inhibitors plus chemo- or radiotherapy may probably display much better outcome in pancreatic cancer patients (44).

Furthermore, CSCs refer to a group of rare immortal cells that could maintain clones of continuously growing tumors (88). The stem-cell frequency in a cancer is correlated with prognosis and therefore, targeting CSCs through m^6^A regulation might be beneficial. For instance, competitive antagonists inhibiting ALKBH5 over other AlkB subfamily proteins such as FTO could possibly reduce the enrichment of BCSCs and impair their ability to initiate breast tumor (62). Nevertheless, m^6^A regulatory enzymes might exert distinct impact on stem cells in physiological and pathological conditions as mentioned above (42, 43, 69, 73, 79). It is plausible to put forward that the m^6^A-targeted strategy in CSCs must be conducted on the premise of distinguishing the normal stem cells from CSCs.

## Conclusion

Evidently, m^6^A modification has tremendous influence on RNA life cycle including RNA processing, nuclear export, and translation or degradation. At the same time, m^6^A is involved in biological processes such as stem cell maintenance, tissue differentiation, and immune response. It seems that cellular m^6^A levels need to be kept within an optimal range, whereas aberrant expression of m^6^A factors will lead to cancer progression. Scientists have explored the impact of m^6^A modification on gene expression and altered cell phenotypes, in hope of presenting novel approaches to conquer diseases.

However, clinical practice of small-molecular inhibitors targeting enzymes modulating m^6^A levels has a great prospect but is still in its infancy. Several issues need to be tackled for the realization of its full potential. A major problem is that we need to gain a better understanding of the selectivity in transcripts and methylated sites in various tissues. Methylation patterns on transcripts might be molecular markers, which recruit distinct m^6^A readers to enter downstream metabolism, respectively. Subsequently, side effects caused by the complex mRNAs targeted by m^6^A enzymes may prevent the agents from achieving a favorable therapeutic index in the clinic. Moreover, heterogeneity in human cancer gives rise to distinct karyotypic patterns, protein and biomarker levels, and genetic profiles, which also requires consideration (89).

In conclusion, molecular mechanism of m^6^A regulation in cancer biology still requests further exploration. Future researches could be focused on seeking the general discipline of specific interaction between m^6^A mark and reader proteins as well as the heterogeneity in distinct tumor origins. Undoubtedly, m^6^A methylation harbors great potential in exploiting brand-new therapies for human cancers. In the future, combination of small-molecule inhibitors targeting m^6^A modification, biological agents, and immunotherapies may improve patient outcomes.

## Author Contributions

SY and XLi wrote the first draft of the manuscript. SL organized the structure of the manuscript. RY, XLiu, and SW contributed conception of the work. All authors contributed to manucript revision, read, and approved the submitted version.

### Conflict of Interest

The authors declare that the research was conducted in the absence of any commercial or financial relationships that could be construed as a potential conflict of interest.
